# Alterations in arthropod and neuronal exosomes reduce virus transmission and replication in recipient cells

**DOI:** 10.20517/evcna.2022.30

**Published:** 2022-08-31

**Authors:** Kehinde Damilare Fasae, Girish Neelakanta, Hameeda Sultana

**Affiliations:** Department of Biomedical and Diagnostic Sciences, College of Veterinary Medicine, University of Tennessee, Knoxville, TN 37996, USA.

**Keywords:** Exosomes, neurons, skin cells, tick, Langat virus, transmission, treatments

## Abstract

**Aim:**

Targeting the modes of pathogen shedding/transmission via exosomes or extracellular vesicles has been envisioned as the best approach to control vector-borne diseases. This study is focused on altering exosomes stability to affect the pathogen transmission from infected to naïve recipient cells.

**Methods:**

In this study, neuronal or arthropod exosomes were treated at different temperatures or with different salts or pH conditions to analyze their ability and efficiency in the transmission of tick-borne Langat virus (LGTV) from infected to naïve recipient cells.

**Results:**

Quantitative real-time PCR (qRT-PCR) and immunoblotting analyses revealed that treatment of neuronal or tick exosomes at warmer temperatures of 37 °C or 23 °C, respectively, or with sulfate salts such as Magnesium or Ammonium sulfates or with highly alkaline pH of 9 or 11.5, dramatically reduced transmission of LGTV via infectious exosomes (human or tick cells-derived) to human neuronal (SH-SY5Y) cells or skin keratinocytes (HaCaT cells), respectively.

**Conclusion:**

Overall, this study suggests that exosome-mediated viral transmission of vector-borne pathogens to the vertebrate host or the viral dissemination and replication within or between the mammalian host can be reduced by altering the ability of exosomes with basic changes in temperatures, salts or pH conditions.

## INTRODUCTION

Exosomes are membrane-bound small vesicles that have become the highlight of research in infectious diseases^[1–12]^. Several viruses perhaps use exosomes [small extracellular vesicles (EVs) of 30–120 nm, with infectious cargo] for transmission of viral RNA/proteins to the naïve recipient host cells^[2–4,13–22]^. Our previous studies have shown that arthropod-borne flavivirus full-length RNA genomes and proteins [such as Envelope protein, Non-Structural protein 1 (NS1) and/or polyprotein] are transmitted to the vertebrate host cells via arthropod exosomes^[1,3,4,23]^. Flaviviral transmission from the infected vectors (such as ticks and mosquitoes) to the vertebrate host via exosomes could be an important strategy for the dissemination of these vector-borne pathogens^[3,4]^. In our previous study, a proof of principle concept illustrates that neuroinvasion of flaviviruses is perhaps mediated via extreme production, release and dissemination of arthropod exosomes through saliva, that fuses with skin barrier cells such as keratinocytes, thus leading to virus dissemination into the peripheral system. High viral loads or viremia in the periphery such as blood, spleen, and liver may breach the blood-brain barrier (BBB) integrity, therefore leading to infection of the CNS^[3,4]^. Our novel line of discovery further led us to isolate exosomes from saliva and salivary glands of hard ticks such as *Ixodes scapularis* and *Amblyomma maculatum*^[23,24]^. Tick salivary and saliva-derived exosomes showed an immune regulation at the vertebrate skin interface by inhibition of human skin C-X-C motif chemokine ligand (CXCL-12) and induction of Interleukin-8 (IL-8) chemokine^[24]^. This immune regulation dramatically delayed cell migration, wound healing and repair process to support continuous and successful blood feeding at the tick bite site^[24]^. Detection of exosomes in tick saliva and salivary glands indeed suggested a potential route of pathogen transmission from the infectious vector to the naïve vertebrate host^[3,24]^. In both these studies, we have characterized *I. scapularis* tick saliva, salivary gland and ISE6 tick cell-derived exosomes^[3,24]^. Given the importance of pathogen transmission via arthropod exosomes to the vertebrate host, it is not surprising to envision that targeting arthropod exosomes could be the best transmission-blocking strategy to interfere and control vector-borne diseases.

Arthropod-borne flaviviruses are transmitted by the bite of an infected vector such as *I. scapularis* ticks which are obligate hematophagous parasites^[3,23,25–31]^. The hard ticks, vector/transmit several human pathogens, including tick-borne encephalitis virus (TBEV), Powassan virus (POWV) and Langat virus (LGTV) in the genus flavivirus^[25–31]^. There are no known cases of human diseases associated with LGTV, and it does not pose a significant epidemiological threat in comparison to TBEV/POWV^[25–31]^. LGTV is used as a model pathogen to study the pathogenesis of deadly pathogens such as TBEV and POWV. During blood feeding, ticks secrete saliva that contains various anti-inflammatory, anti-coagulatory, anti-vasoconstrictory and anti-platelet aggregation factors, which regulate vertebrate host immune responses^[32–39]^. Ticks are fed for a prolonged period of time which could be up to 2 weeks. During this lengthy feeding time, ticks must encounter the vertebrate host defense responses to get a complete blood meal, while at the same time also transmitting pathogens that need to evade, survive, replicate, and colonize in the host. At the tick feeding site, there is a triad interaction that involves pathogens, ticks and their vertebrate hosts^[32–39]^. Being a key regulator of the triad interactions, the pathogen modulates tick feeding time, efficiency of tick feeding and salivary components/saliva secretion^[1,23,40]^. It is noteworthy that ticks co-exist with several pathogens and have been evolving with them for over a period of time. This co-evolution with pathogens perhaps adapts ticks with better strategies to overcome host defense mechanisms by modulating the saliva components with various pharmacologically active molecules and exosomes that could inhibit immune responses at the vertebrate skin interface. These salivary modulations could be pathogen directed and favorable to the vector host in order to co-exist and benefit each other from this long-term relationship. There are several studies including transcriptomics/proteomics and sialomics, with evidence that tick-transmitted pathogens manipulate the salivary/saliva components to facilitate their transmission, evade host defense reactions and survive in the vertebrate host^[35–39,41–47]^. Our findings revealed that tick exosomes are salivary/saliva components and that they do facilitate pathogen transmission to the vertebrate host, suggesting that they are novel means of the dissemination from vector to the vertebrate host^[3,24]^. Treatment of arthropod/neuronal exosomes with GW4869 (a pharmacological agent that interferes with the production and release of exosomes) inhibits the viral RNA/proteins and also interferes with the transmission of flaviviruses from exosomes to the naïve recipient host cells^[2–4,25,48,49]^. The mechanism(s) of GW4869 affecting viral transmission has not been clearly understood. In this study, we have performed some basic treatments of exosomes at different temperatures with different salts or pH conditions to understand their stability and efficiency in the transmission and replication of tick-borne viruses.

## METHODS

### Cell lines, cell culture and viral infections.

Human neuroblastoma cells (SH-SY5Y; CRL-2266; American Type culture Collection: ATCC), and *Ixodes scapularis* tick embryonic (ISE_6_; NR12234; BEI Resources) cell lines were purchased from ATCC/BEI and propagated as per the instructions from the supplier. Human SH-SY5Y cells were grown in complete EMEM medium (ATCC recommended) with 10% heat-inactivated Fetal bovine serum (FBS) (SIGMA) at 37 °C and 5% CO_2_, respectively. ISE6 cells were cultured and grown in L15B300 medium (SIGMA) at 34 °C and as per the recommendations from Dr. Munderloh^[50,51]^. Human skin keratinocytes (HaCaT cells) were obtained from Addexbio Technologies/FisherScientific and grown (as per the instructions from the supplier) in complete Dulbecco’s Modified Eagle Medium (DMEM; SIGMA) with 10% heat-inactivated FBS at 37 °C and 5% CO_2_. SH-SY5Y or ISE6 cells were plated at densities of 1–2 × 10e6, and after overnight incubations, cells were infected with Langat virus [LGTV, strain LGT-TP21, 1 MOI (multiplication of infection) for ISE6 cells or 5 MOI for SH-SY5Y cells]. Naïve SH-SY5Y or HaCaT cells were infected via infectious exosomes collected from either SH-SY5Y or tick cells, respectively.

### Isolation of exosomes from infected cell culture supernatants and treatment at different temperatures, salts or pH conditions.

Exosomes were isolated from the independent batch of LGTV-infected (72 h postinfection, p.i.) human SH-SY5Y or ISE6 tick cell culture supernatants and by differential ultracentrifugation method as previously described^[2–4]^. Exosome pellets were resuspended in 300–350 μl of appropriate solutions, depending on the respective treatments and as described in Table 1. Exosome suspensions were aliquoted (as equal volumes) into respective solutions and held overnight at 4 °C (in case of pH or salt treatments) or at respective temperatures (of −80 °C, 4 °C, 12 °C, 23 °C or 37 °C) in 1 × chilled phosphate buffered saline solution (PBS; SIGMA) and as indicated in Table 1. Exosomes collected after pelleting were directly suspended in 1 × chilled PBS (with neutral pH of 7.0 for the temperature group) or in 1 × chilled PBS (tubes were adjusted to achieve respective pH of 1.5, 4, 7, 9 or 11.5). In this study, we used 1 × PBS (SIGMA) with neutral pH of 7, which is adjusted to make either acidic or basic solutions of respective pH. Also, we used 1 × chilled PBS (with neutral pH of 7.0) to prepare 0.1 M (each one) respective salt solutions [of Sodium Chloride, NaCl; Ammonium sulfate, (NH_4_)_2_SO_4_; Sodium Acetate, CH_3_COONa or Magnesium Sulfate, MgSO_4_]. Each treated group of exosomes from SH-SY5Y or ISE6 tick cells was independently prepared (batch by batch) and processed for independent re-infection on naïve recipient cells (SH-SY5Y or HaCaT cells), respectively. Recipient cells (human SH-SY5Y or HaCaT) that received infectious and treated neuronal or tick exosomes, respectively, were all incubated at 37 °C and 5% CO_2_. A detailed schematic representation is shown for experimental procedures and treatments [Supplementary Figure 1].

### Infection of naïve SH-SY5Y or HaCaT cells via infectious exosomes treated at different temperatures, salts or pH conditions.

For determining the ability and efficiency of infectious exosomes (treated with respective temperatures, salts or pH) to transmit LGTV, we used human SH-SY5Y cells as recipient host cells for neuronal exosomes (prepared from the independent batch of SH-SY5Y cells) or HaCaT cells as recipient host cells for exosomes derived from tick ISE6 cells. Naïve SH-SY5Y or HaCaT recipient cells (at a density of 1–2 × 10e5) were seeded per well and as 5–6 replicates per treatment (5 replicates for RNA extractions and 1 sample for protein extraction, respectively). Recipient cells were plated on the same day of exosome isolation, and allowed to adhere while exosomes were independently incubated overnight at respective treatments. Following overnight treatments, exosomes treated with respective conditions were incubated (20 μL of exosome solution per replicate per treatment/group) on naïve SH-SY5Y or HaCaT cells, respectively. After three days of post-incubation with infectious exosomes, SH-SY5Y or HaCaT recipient cells were lysed in either RLT lysis buffer or modified RIPA lysis buffer for extractions of total RNA or proteins, respectively. A detailed schematic representation is shown for experimental procedures and treatments [Supplementary Figure 1].

### Total RNA extractions, cDNA synthesis and qRT-PCR analysis.

RNA extractions were performed using Aurum Total RNA Mini kit (Bio-Rad) and by following the manufacturer’s instructions. Total RNA from SH-SY5Y or HaCaT cells was eluted in small volumes (50 μl) of Nuclease free water. RNA was converted to cDNA using the iScript cDNA synthesis kit (Bio-Rad). The generated cDNA was used as the template for the amplification and determination of viral loads in the recipient cells. We used previously published primers for the detection of LGTV PrM transcripts^[3,4]^. To normalize the amount of template, human beta actin amplicons were quantified using previously published primers^[3,4]^. qRT-PCR was performed using iQSYBR Green Supermix (Bio-Rad). Standard curves were prepared using 10-fold serial dilutions starting with standards 1 to 6 of known quantities of actin or LGTV fragments. q-PCR reactions were performed using CFX Opus instrument (Bio-Rad) and as previously described^[3,4]^.

### Immunoblotting analysis.

To determine the viral loads, 10–20 μg of total protein from respective cell lysates (either SH-SY5Y or HaCaT cells incubated with neuronal or tick exosomes, treated at different temperatures, salts or pH conditions) were resolved on 12% SDS-PAGE gels. Following gel electrophoresis, Western blotting was performed as described^[3,4]^. Blots were blocked with 5% milk buffer and probed with 6E11 anti-Langat monoclonal primary antibody (at 1:1000 dilution; BEI resources) to detect NS1 protein, followed by incubation with mouse HRP-conjugated secondary antibody (at 1:5000 dilution; Boster Biological Technology). Either samples were re-run separately (for SH-SY5Y cells), or blots were re-probed (for HaCaT cells) with anti-CD9 or anti-CD63 monoclonal antibodies, respectively, (Novus Biologicals, LLC), followed by their respective secondary antibodies. Total protein profile gel images were obtained from stain-free gels. Antibody binding was detected with the WesternBright ECL kit. Blots/gels were imaged using the Chemidoc MP imaging system and processed using Image Lab software obtained from the manufacturer (Bio-Rad).

### Statistical analyses.

Statistical significance of differences observed in data sets was analyzed using GraphPad Prism 6 software and Microsoft Excel. The unpaired, two-tailed Student *t-*test was used for all analyses. Error bars represent mean (± SEM) values and Standard Error. Also, *P* values < 0.05 were considered significant in all analyses.

## RESULTS

### Neuronal exosomes infected with LGTV showed the presence of CD9 and HSP70 exosomal markers.

We first tested infected neuronal exosomes in this study, since LGTV is very similar to TBEV and POWV neuroinvasive viruses that cause neuropathogenesis, neuroinflammation, neuronal loss, and encephalitis that ultimately leads to death. Exosomes isolated from human SH-SY5Y cells were processed for detection of exosomal markers such as cell surface glycoprotein, CD9 (a member of the tetraspanins family) and the heat shock protein 70 (HSP70), a component of exosomal lumen. Immunoblotting analysis revealed that both CD9 and HSP70 were detected in LGTV-infected SH-SY5Y total exosomal-protein lysates [Supplementary Figure 2]. It was noted that both CD9 and HSP70 were upregulated in LGTV-infected group in comparison to the respective uninfected control groups [Supplementary Figure 2]. Immunoblotting analysis using LGTV-6E11 antibody revealed detection of viral NS1 in infected groups. Total protein profile gel images serve as loading controls [Supplementary Figure 2].

### Neuronal exosomes treated at cold temperatures transmit increased LGTV loads to naïve recipient cells.

**Figure 1.**
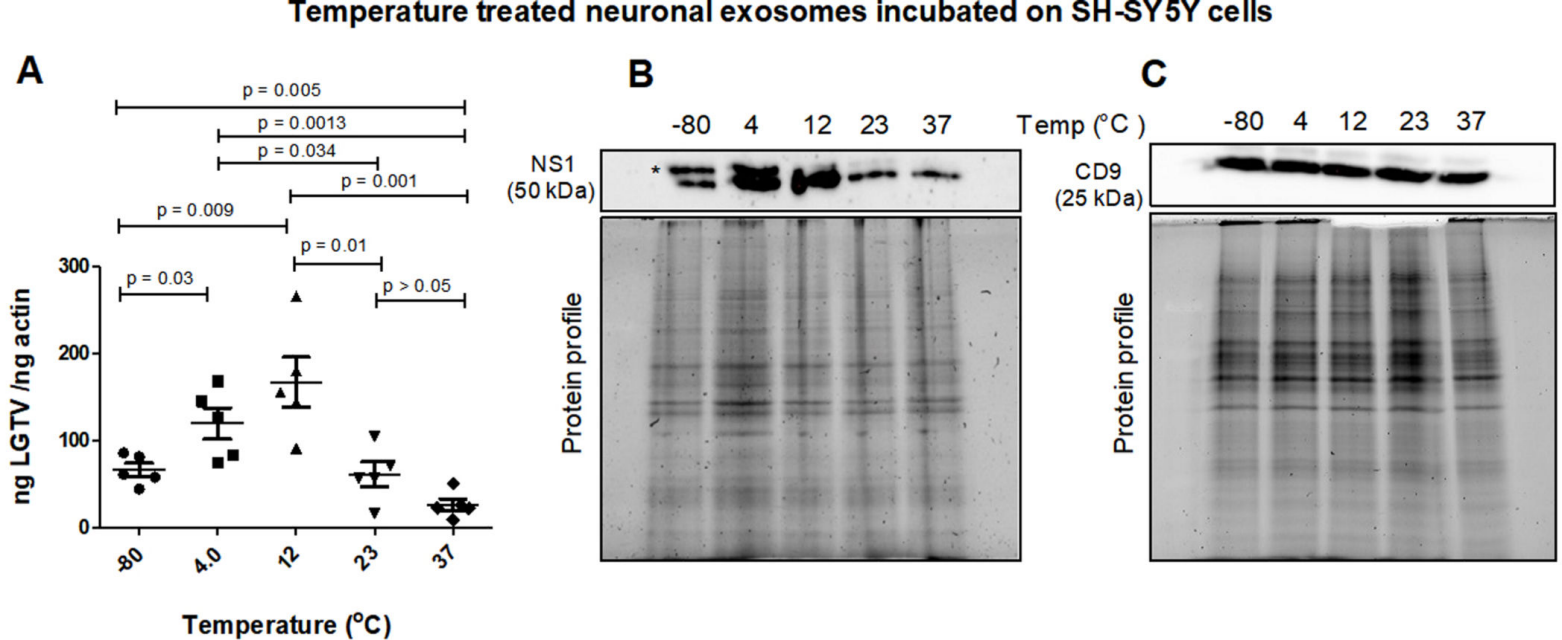
(A) qRT-PCR analysis showing viral burden (PrM transcript levels) from SH-SY5Y recipient cells that were incubated with infectious neuronal exosomes treated at varying temperatures (of −80 °C, 4 °C, 12 °C, 23 °C, or 37 °C) and held overnight in 1 × chilled PBS (of pH 7). All temperatures are represented as degree Celsius. Closed circles, squares, triangles, inverted triangles, or rhombus represents viral loads detected in LGTV-infected SH-SY5Y recipient cells incubated with infectious exosomes treated at indicated temperatures. In panel A, each data point represents data from one independent culture well in a plate. Each treatment had five independent replicates. The mRNA levels of *prM* gene are normalized to human beta-actin mRNA levels. *P* values less than 0.05 or equal to the numbers shown on the graph are from Student’s *t*-test. There is no statistical difference between the data observed in −80 °C and 23 °C or 4 °C and 12 °C groups. Immunoblotting analysis is shown for proteins NS1 (B) or CD9 (C) at all tested temperatures. Asterisk (*) indicates glycosylated NS1 protein. Protein sizes are indicated in kilodaltons (kDa). Protein profile gel images in (B) and (C) are shown as loading controls.

Neuronal exosomes derived from LGTV-infected human SH-SY5Y cells, incubated overnight in PBS and at different temperatures (of −80 °C, 4 °C, 12 °C, 23 °C or 37 °C) were allowed to transmit viral loads to naïve human recipient SH-SY5Y cells. After 3-day post-incubations, LGTV loads were determined in recipient cells incubated with infectious exosomes treated at different temperatures [Figure 1A]. qRT-PCR analysis revealed significantly (*P* < 0.05) increased LGTV loads in SH-SY5Y cells incubated with exosomes treated at cold temperatures (of 4 °C or 12 °C) compared to the viral loads noted from cells incubated with exosomes treated at other temperatures (of −80 °C, 23 °C or 37 °C) [Figure 1A]. SH-SY5Y cells incubated with exosomes treated at a higher temperature of 37 °C (which is also the human body temperature) had significantly lower viral loads in comparison to viral loads noted in cells incubated with exosomes treated at 23 °C (room temperature), or 12 °C (cold) or freezing temperature of −80 °C [Figure 1A]. Similar results were obtained with the immunoblotting analysis where LGTV NS1 protein loads were increased in SH-SY5Y cells incubated with exosomes treated at cold temperatures (of 4 °C or 12 °C) when compared to the viral loads noted in cells incubated with exosomes treated at other tested temperatures (of −80 °C, 23 °C or 37 °C) [Figure 1B]. LGTV loads noted in SH-SY5Y cells incubated with exosomes treated at freezing temperature of −80 °C were nearly 2-fold higher or 4-fold enhanced at 4 °C or 12 °C respectively, when compared to viral loads noted in cells incubated with exosomes treated at 23 °C or 37 °C temperatures [Figure 1B]. We also noted the presence of enhanced glycosylated NS1 product in SH-SY5Y cells incubated with exosomes treated at cold temperatures of −80 °C, 4 °C or 12 °C. The NS1 glycosylation product was dramatically lower or faded in cells incubated with exosomes treated at higher temperatures of 23 °C or 37 °C [Figure 1B]. Human CD9 (an exosomal marker protein) loads showed no differences in any samples [Figure 1C]. Total protein profile gel images serve as loading controls [Figure 1B and C].

### Neuronal exosome-mediated LGTV transmission is inhibited by both Ammonium or Magnesium Sulfates.

**Figure 2.**
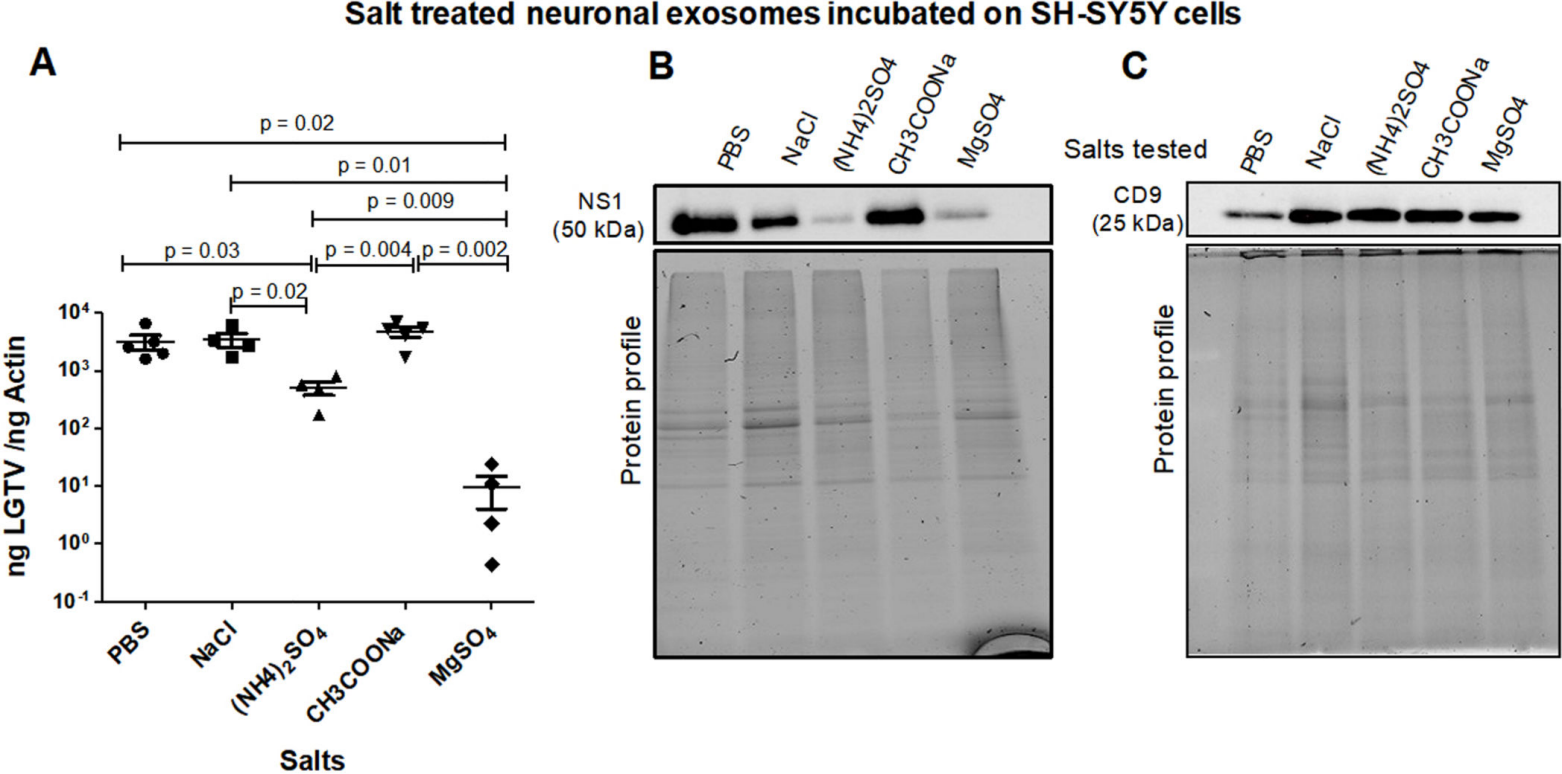
(A) qRT-PCR analysis showing viral loads (PrM transcript levels) from SH-SY5Y recipient cells incubated (for three days) with infectious neuronal exosomes, treated overnight at 4 °C in 1 × chilled PBS (as control) or with PBS enriched 0.1 M salt solutions [of NaCl, (NH_4_)_2_SO_4_, CH_3_COONa or MgSO_4_], respectively. Closed circles, squares, triangles, inverted triangles or rhombus represents viral loads obtained from LGTV-infected SH-SY5Y recipient cells incubated with infectious exosomes treated with respective salts. In panel A, each data point represents data from one independent culture well in a plate. Each treatment had five independent replicates. The mRNA levels of *prM* gene are normalized to human beta-actin mRNA levels. *P* values less than 0.05 or equal to the numbers shown in the graph are from Student’s *t*-test. There is no statistical difference between PBS to NaCl/CH3COONa, or NaCl to CH3COONa group. Immunoblotting analysis is shown for proteins NS1 (B) or CD9 (C) and for all tested salts. Protein sizes are indicated in kilodaltons (kDa). Protein profile gel images in (B) and (C) are shown as loading controls.

LGTV-infectious exosomes derived from human SH-SY5Y cells, incubated overnight at 4 °C in 1 × chilled PBS (as control) or with PBS enriched 0.1 M salt solutions [of NaCl, (NH_4_)_2_SO_4_, CH_3_COONa or MgSO_4_], respectively, were incubated on naïve recipient SH-SY5Y cells for 3 days. Treatments with all salt solutions allowed LGTV transmission via infectious exosomes, and replication in recipient cells [Figure 2A]. qRT-PCR analysis revealed that exosomes incubated in PBS, NaCl or CH3COONa demonstrated significantly high LGTV loads when compared to viral loads observed in cells incubated with exosomes treated with (NH_4_)_2_SO_4_ or MgSO_4_ [Figure 2A]. Between these two sulfate treatments, SH-SY5Y cells incubated with exosomes treated with MgSO_4_ showed significantly lower viral loads when compared to cells incubated with exosomes treated with (NH_4_)_2_SO_4_ [Figure 2A]. Immunoblotting analysis of these samples also demonstrated lower NS1 protein loads in SH-SY5Y cells incubated with exosomes treated with (NH_4_)_2_SO_4_ or MgSO_4_ when compared to the other salt treated groups [Figure 2B]. NS1 protein nearly showed 4–5 folds reduction in SH-SY5Y cells incubated with exosomes that were treated with (NH_4_)_2_SO_4_ or MgSO_4_ [Figure 2B]. Human CD9 loads showed no differences between any salt treatments; however, lower loads of CD9 in PBS control is perhaps due to loading differences [Figure 2C]. Total protein profile gel images serve as loading controls [Figure 2B and C].

### Neuronal exosome-mediated LGTV transmission is drastically reduced upon treatment at alkaline pH.

**Figure 3.**
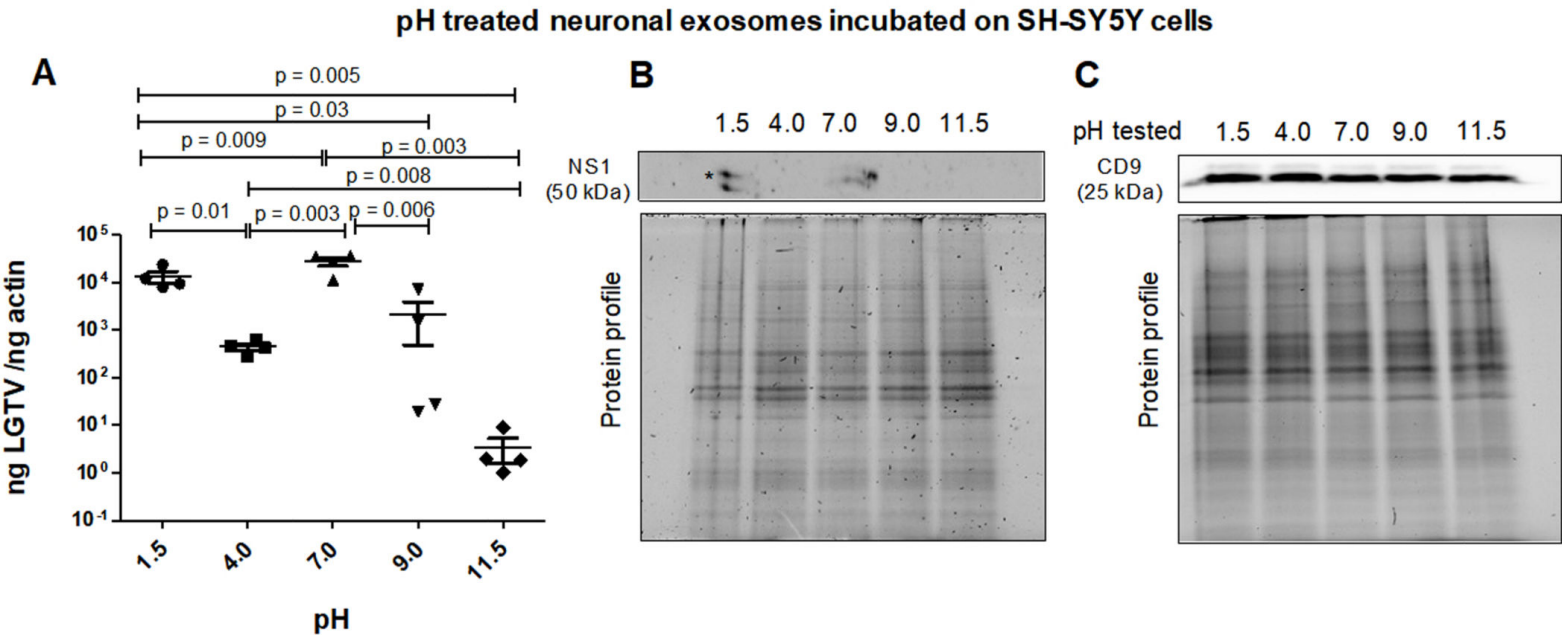
(A) qRT-PCR analysis showing viral loads (PrM transcript levels) from SH-SY5Y recipient cells that were incubated (for three days) with infectious neuronal exosomes, pretreated overnight at 4°C and with different pH conditions (of 1.5, 4, 7, 9 or 11.5), respectively. All pH conditions were set in 1x PBS solution. Closed circles, squares, triangles, inverted triangles or rhombus represents viral loads obtained from LGTV-infected SH-SY5Y recipient cells incubated with infectious exosomes treated at indicated pH. In panel A, each data point represents data from one independent culture well in a plate. Each treatment had five independent replicates. The mRNA levels of *prM* gene are normalized to human beta-actin mRNA levels. *P* values less than 0.05 or equal to the numbers shown in the graph are from Student’s *t*-test. There is no statistical difference between groups of pH 4 and 9. Immunoblotting analysis is shown for proteins NS1 (B) or CD9 (C) and for all tested pH conditions. Asterisk (*) indicates glycosylated NS1 protein. Protein sizes are indicated in kilodaltons (kDa). Protein profile gel images in (B) and (C) are shown as loading controls.

Neuronal exosomes (derived from LGTV-infected SH-SY5Y host cells) were incubated overnight at 4 °C in PBS solutions with varying pH (of 1.5, 4, 7, 9, or 11.5) and then applied to naïve recipient SH-SY5Y cells for three days post-incubations. It was noted that between the tested pH conditions, both acidic pH (of 1.5 or 4) and the neutral pH of 7-treated infectious exosomes enhanced viral transmission and replication in recipient cells [Figure 3A]. qRT-PCR analysis showed significantly lower LGTV loads in SH-SY5Y cells incubated with exosomes that were treated at alkaline pH of 9 or 11.5 in comparison to the viral loads noted in cells incubated with exosomes that were treated at acidic (of 1.5 or 4) or neutral pH (of 7) [Figure 3A]. Between the two alkaline pH treatments, SH-SY5Y cells incubated with exosomes treated at pH of 11.5 showed significantly lower LGTV loads when compared to viral loads noted in cells incubated with exosomes treated at pH of 9. In contrast, SH-SY5Y cells incubated with exosomes treated at acidic pH of 1.5 showed higher LGTV loads when compared to viral loads noted in cells incubated with exosomes treated at acidic pH of 4 [Figure 3A]. Immunoblotting analysis further confirmed the qRT-PCR data and showed the presence of NS1 in only SH-SY5Y cells incubated with exosomes treated at acidic pH of 1.5 or neutral pH of 7 [Figure 3B]. NS1 protein was not detected in SH-SY5Y cells incubated with exosomes treated at alkaline pH (of 9 or 11.5) or acidic pH of 4 [Figure 3B]. No differences were observed in human CD9 protein levels in all tested samples [Figure 3C] and total protein profile gel images serve as loading controls [Figure 3B and C].

### Tick exosomes treated at cold temperatures transmit increased LGTV to human skin cells.

**Figure 4.**
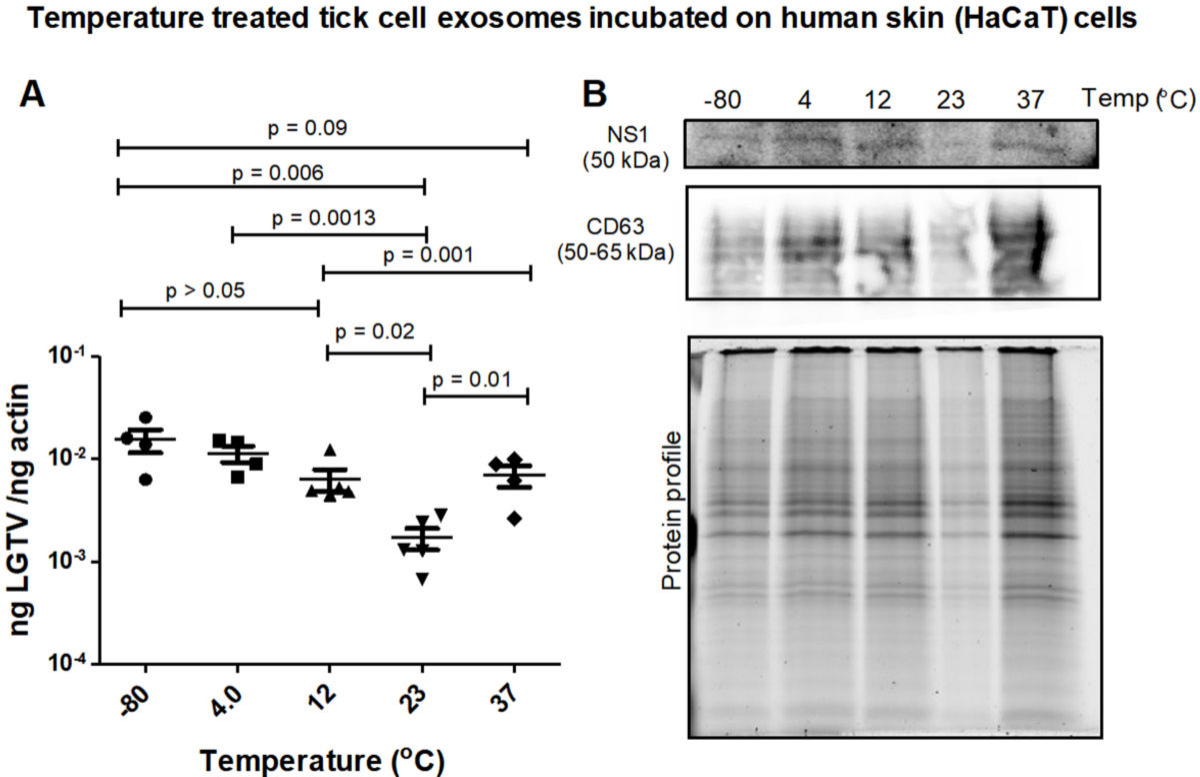
(A) qRT-PCR analysis showing viral loads (PrM transcript levels) from HaCaT recipient cells incubated with infectious tick exosomes treated at varying temperatures (of −80 °C, 4 °C, 12 °C, 23 °C, or 37 °C), and held overnight in 1 × chilled PBS with pH 7. All temperatures are represented as degree Celsius. Closed circles, squares, triangles, inverted triangles or rhombus represents viral loads obtained from LGTV-infected HaCaT cells incubated with infectious tick exosomes treated at indicated temperatures. In panel A, each data point represents data from one independent culture well in a plate. Each treatment had five independent replicates. The mRNA levels of *prM* gene are normalized to human beta-actin mRNA levels. *P* values less than 0.05 or equal to the numbers shown in the graph are from Student’s *t*-test. There is no statistical difference between −80 °C and 4 °C, or 4 °C to 12 °C and 37 °C groups. Immunoblotting analysis is shown for proteins NS1 or CD63 (B) at all tested temperatures. Protein sizes are indicated in kilodaltons (kDa). Protein profile gel image in (B) is shown as the loading control.

LGTV-infected exosomes derived from ISE6 tick cells, held overnight in 1 × chilled PBS solution and at varying temperatures (of −80 °C, 4 °C, 12 °C, 23 °C or 37 °C), were incubated on naïve recipient human skin keratinocytes (HaCaT cells) for three days. LGTV transmission (via infectious tick exosomes) and replication in recipient HaCaT cells were detected in all tested temperatures [Figure 4A]. qRT-PCR analysis showed that HaCaT cells had significantly (*P* < 0.05) increased LGTV loads when incubated with tick exosomes treated at freezing/refrigeration temperatures (of −80 °C, 4 °C or 12 °C) when compared to the viral loads noted in cells incubated with tick exosomes treated at warmer temperatures of 23 °C or 37 °C [Figure 4A]. Immunoblotting analysis also revealed that tick exosomal mediated viral transmission to HaCaT cells showed lower replication of LGTV when recipient cells were incubated with tick exosomes treated at 23 °C [Figure 4B]. However, higher viral loads were noted in recipient HaCaT cells incubated with tick exosomes treated at cold temperatures (of 4 °C or 12 °C) [Figure 4B]. Human CD63 loads were detected in all tested samples and total protein profile gel image serves as the loading control [Figure 4B].

### Tick exosome-mediated LGTV transmission is inhibited by Magnesium Sulfate.

**Figure 5.**
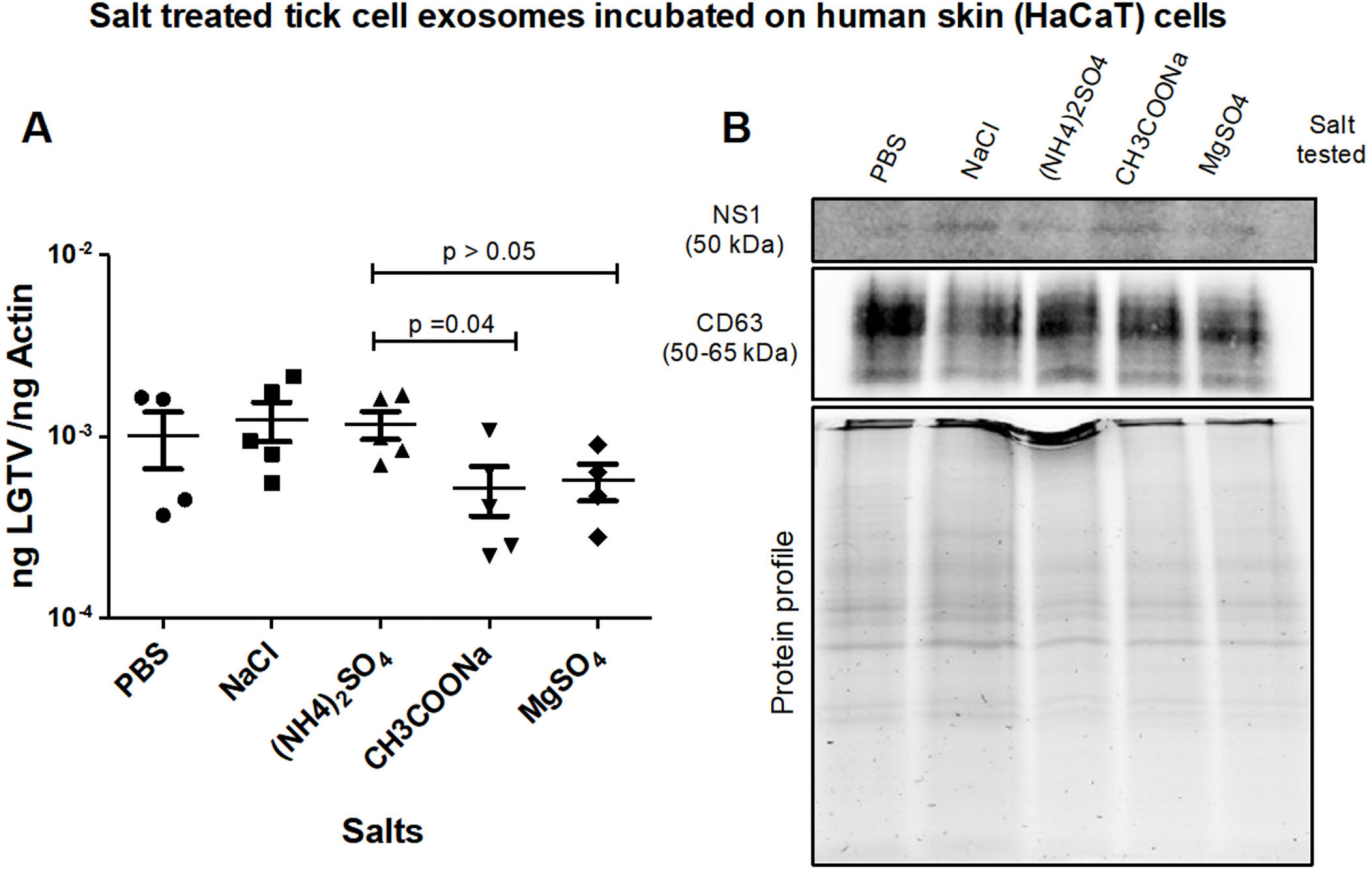
(A) qRT-PCR analysis showing viral loads (PrM transcript levels) from HaCaT recipient cells that were incubated (for three days) with infectious tick exosomes, pretreated overnight at 4 °C in 1 × chilled PBS (as control) or with PBS enriched 0.1 M salt solutions [of NaCl, (NH_4_)_2_SO_4_, CH_3_COONa or MgSO_4_], respectively. Closed circles, squares, triangles, inverted triangles or rhombus represents viral loads obtained from LGTV-infected HaCaT cells incubated with infectious tick exosomes treated with indicated salts. In panel A, each shape represents data from one independent culture well in a plate. Each treatment had five independent replicates. The mRNA levels of *prM* gene are normalized to human beta-actin mRNA levels. *P* values less than 0.05 or equal to 0.04 are from Student’s *t*-test. There is no statistical difference between PBS/NaCl and other tested salts, or CH_3_COONa and MgSO_4_ groups. Immunoblotting analysis is shown for proteins NS1 or CD63 (B) and for all tested salts. Protein sizes are indicated in kilodaltons (kDa). Protein profile gel image in (B) is shown as the loading control.

LGTV-infectious exosomes derived from ISE6 tick cells, held overnight at 4 °C in PBS or PBS enriched 0.1 M salt solutions [of NaCl, (NH_4_)_2_SO_4_, CH_3_COONa or MgSO_4_], respectively, were incubated on naïve recipient HaCaT cells for 3 days. All tested salt solutions allowed LGTV transmission and viral replication in HaCaT cells [Figure 5A]. qRT-PCR analysis revealed that HaCaT cells incubated with tick exosomes treated with PBS (control), NaCl or (NH_4_)_2_SO_4_ demonstrated significantly higher LGTV loads when compared to the viral loads detected in cells incubated with tick exosomes treated with CH_3_COONa or MgSO_4_ [Figure 5A]. Immunoblotting analysis further supported the qRT-PCR data [Figure 5B]. No differences in CD63 levels were noted in all tested samples [Figure 5B]. Total protein profile gel image serves as the loading control [Figure 5B].

### Tick exosomes treated at alkaline pH conditions transmit reduced LGTV loads to human skin cells.

**Figure 6.**
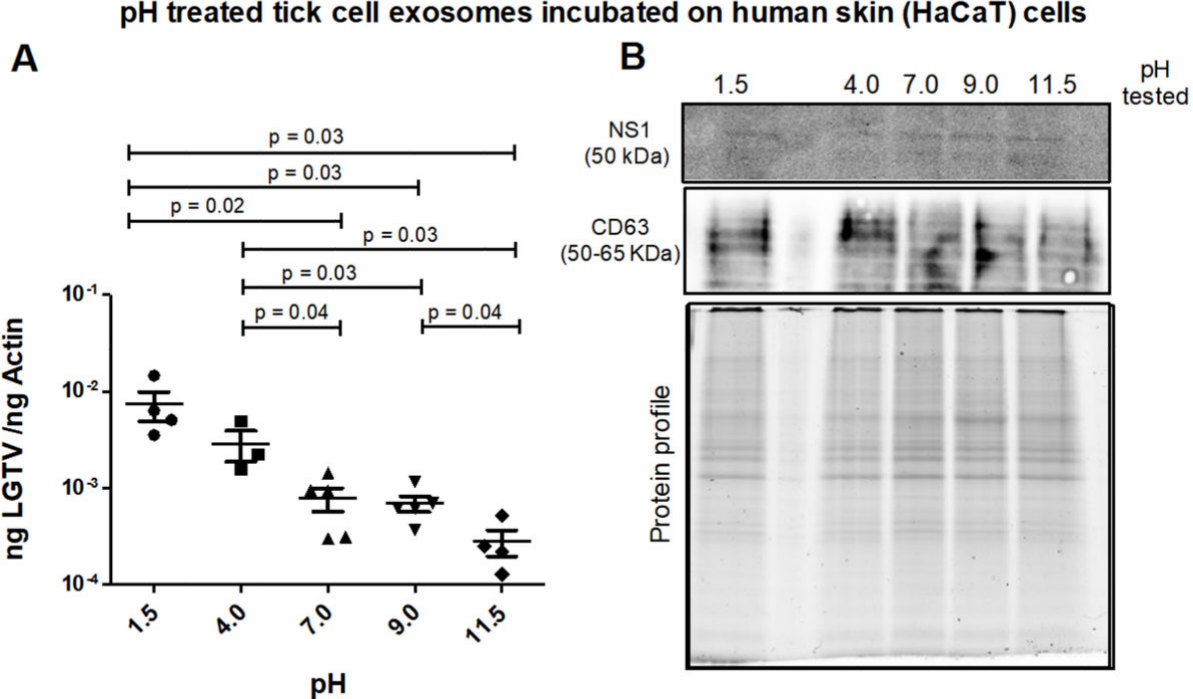
(A) qRT-PCR analysis showing viral loads (PrM transcript levels) from HaCaT recipient cells that were incubated (for 3 days) with infectious tick exosomes, treated overnight at 4 °C and at different pH conditions (of 1.5, 4, 7, 9 or 11.5), respectively. All pH conditions were set in 1x PBS solution. Closed circles, squares, triangles, inverted triangles or rhombus represents viral loads obtained from LGTV-infected HaCaT cells incubated with infectious exosomes treated at indicated pH. In panel A, each data point represents data from one independent culture well in a plate. Each treatment had five independent replicates. The mRNA levels of *prM* gene are normalized to human beta-actin mRNA levels. *P* values less than 0.05 or equal to the numbers shown in the graph are from Student’s *t*-test. There is no statistical difference between groups of pH 1.5 and 4, or 7 to 9 and 11.5. Immunoblotting analysis is shown for proteins NS1 or CD63 (B) and for all tested pH conditions. Protein sizes are indicated in kilodaltons (kDa). Protein profile gel image in (B) is shown as the loading control.

Infectious tick exosomes (derived from LGTV-infected tick cells) were incubated overnight at 4 °C in PBS solutions with varying pH (of 1.5, 4, 7, 9 or 11.5) and then incubated on naïve recipient HaCaT cells for three days. It was noted that between the tested pH conditions, HaCaT cells incubated with tick exosomes treated at both acidic pH (of 1.5 or 4) had enhanced LGTV loads when compared to the viral loads noted in cells incubated with tick exosomes treated at other tested pH conditions (of 7, 9 or 11.5) [Figure 6A]. qRT-PCR analysis showed significantly (*P* < 0.05) lower LGTV loads in HaCaT cells incubated with exosomes treated at alkaline pH (of 9 or 11.5) in comparison to the viral loads noted in cells incubated with exosomes treated at acidic pH (of 1.5 or 4) [Figure 6A]. Between the two alkaline pH treatments, HaCaT cells incubated with tick exosomes treated at pH 11.5 showed significantly lower LGTV loads when compared to viral loads noted in cells treated with exosomes held at pH 9. In addition, HaCaT cells incubated with exosomes treated at neutral pH (of 7) showed lower LGTV loads when compared to viral loads in cells incubated with exosomes treated at acidic pH (of 1.5 or 4) [Figure 6A]. Immunoblotting analysis showed the presence of NS1 in all tested pH conditions [Figure 6B]. CD63 protein was detected in all analyzed samples and total protein profile gel image serves as the loading control [Figure 6B].

## DISCUSSION

Arthropod-borne flaviviruses that are positive-sense single-stranded RNA viruses usually cycle between the vector and natural reservoir host but can accidentally infect human and other vertebrate hosts^[52–55]^. Some of the flaviviruses are emerging or re-emerging pathogens that could lead to a large economic burden^[1,23,40]^.

Both developed and developing countries are prone to outbreaks of vector-borne flaviviral infections, their related diseases, and deaths^[1,23,40]^. Currently, there are no approved vaccines or specific therapeutics to target flaviviral infections. We envision targeting the modes of pathogen shedding or transmission as the best approach to control vector-borne diseases. Our previous studies showed that flaviviruses such as tick-borne LGTV or mosquito-borne dengue (DENV), Zika virus (ZIKV) or West Nile virus (WNV) use arthropod exosomes as modes of transmission from vector to the vertebrate host^[2–4,24,25]^. For the first time, our laboratory showed that medically important vectors such as ticks and mosquitoes secrete exosomes in heterogenous populations^[3,4]^. Our studies also showed that both tick- and mosquito-borne flaviviruses profusely use arthropod exosomes for transmission from arthropod cells to the human skin keratinocytes or blood endothelial cells^[3,4]^. We have detected viral RNA/full-length RNA genomes and viral proteins such as Envelope (E), Non-Structural protein 1 (NS1) and perhaps fully assembled polyprotein in arthropod and neuronal exosomes^[2–4]^. We believe that the presence of full-length RNA genomes or polyproteins in exosomes could be sufficient to bring in infection in the vertebrate host, suggesting exosomes as a highly infectious route for viral transmission^[2–4]^. We also detected both positive and negative strands of flaviviral RNA that suggest transmission of both assembled and replicative viruses^[3]^. Moreover, our extensive studies showed that all tested evaluations, including RNaseA/proteinase K, and treatments with highly cross-reactive/neutralizing antibodies (such as 4G2 or ZV-2/16 Abs), did not interfere with the viral transmission, suggesting the presence of viral material inside the lumen of exosomes^[2–4,25]^. These studies suggested that exosomal packed viral RNA genomes and proteins are well secured, highly infectious and replicative in naïve recipient host cells^[2–4,25]^.

The role of exosomes in infectious viral diseases is highlighted, and several other studies have also contributed to providing evidence(s) that exosomes are important means for the transmission of viruses from infected cells to the naïve recipient cells^[8,56–63]^. Our studies showed that GW4869 inhibitor affects flaviviral transmission via arthropod or neuronal exosomes^[2–4,25]^. Some studies have shown the effect of temperature and pH on exosomal integrity and functions^[64–68]^. Understanding the impact of exosome storage is limited, as it can change the size, number, contents, and function of exosomes. A recent report suggests that −80 °C is the most favorable condition for long-term storage of biofluids and exosomes and 4 °C for short-term storage^[69]^. In addition, it has been shown that human saliva-derived exosomes are stable for two months at 4 °C, maintaining their membrane integrity over this long storage period^[70]^. Several studies have discussed the inhibitory effects of salts on virus replication^[71–80]^. Given the importance of pathogen transmission via exosomes, it is essential to explore the ways we can potentially alter the exosomes stability, efficiency and cargo transport ability from the host to the naïve recipient cells. However, there are no reports showing treatment of exosomes via temperature, pH or salt can dramatically affect viral transmission. We took a simple approach to interfere with the exosomal delivery by treating or affecting them with temperatures, salts or pH conditions. This testing was performed on both neuronal or tick exosomes to understand their stability and efficiency in tick-borne viral transmission.

In this study, different temperatures (of −80 °C, 4 °C, 12 °C, 23 °C, or 37 °C) were selected based on the long-term or short-term storage of exosomes at −80 °C or 4 °C, respectively. Ticks are maintained at 23 °C in laboratory conditions and the human body is at 37 °C; therefore, we chose these temperatures in addition to 12 °C which lies between the selected temperatures. Both acidic (1.5 or 4) or basic pH (9 or 11.5) were selected to address viral replication and transmission. Neutral pH is considered as the control. In the case of different salts, we preferred the highly consumed table salt NaCl and other salts such as MgSO_4_, which is taken by several people as dietary supplementation. Sodium acetate (CH_3_COONa) is selected as it is a widely used salt across the industrial sectors. In medical situations, sodium acetate is given intravenously as an electrolyte replenishment as it corrects sodium levels in hyponatremic patients. Since ammonium sulfate is used in the purification of proteins including flaviviral proteins, we also considered this salt. We found that LGTV transmission to human SH-SY5Y cells via infectious neuronal exosomes (treated at various temperatures) is increased when incubated with exosomes treated at 4 °C or 12 °C in comparison to exosomes treated at warmer temperatures (of 23 °C or 37 °C) [Figure 1A]. Similar pattern was also noted in human skin keratinocytes that were incubated with tick exosomes treated with same temperatures (as neuronal exosomes), where cold-treated exosomes (treated at −80 °C, 4 °C or 12 °C), resulted in increased viral transmission and replication in naïve recipient skin cells in comparison to exosomes treated at a warmer temperature of 23 °C. This data indicates that at cold temperatures, perhaps exosomes are highly stable and maintain their membrane integrity to secure the inside/luminal contents, including viral RNA/proteins, that could be efficiently transmitted to the naïve recipient host cells. It is noteworthy that neuronal exosomes treated at a higher temperature of 37 °C resulted in less transmission of LGTV to naïve SH-SY5Y cells. However, tick exosomes treated at a higher temperature of 37 °C did not show a lower trend of viral transmission (in comparison to 23 °C) to human skin cells. This observation suggests that tick exosomes, unlike neuronal exosomes, are more stable for prolonged periods, such as tick blood feeding on a vertebrate host that would eventually result in the delivery of infectious exosomes (via saliva secretion), their transport/fusion and transmission of pathogens to human skin cells. In contrast, the neuron-neuron model had very low LGTV loads at 37 °C, thereby suggesting that human body temperature or its slight elevation, such as fever, may control viral transmission between neurons and within the human brain. We also observed a dramatic reduction in NS1 protein loads in naïve SH-SY5Y cells incubated with EVs held overnight at 37 °C. The glycosylated product (visible as a double band) faded at 23 °C or 37 °C in SH-SY5Y cells, suggesting a change in NS1 stability and perhaps polyprotein disassembly at warmer temperatures. The observation of no differences in the CD9 protein levels further suggested a specific change only for viral NS1 protein at higher temperatures. We assume that it is a potential time-dependent adverse effect in the host body on the ability of exosomes to either interact or be taken up by their target cells efficiently, or that this could be a time-dependent effect in the host body on exosome stability. Further studies are needed to provide some mechanistic speculations as to why tick exosomes do better than neuronal exosomes at the same physiologically relevant temperature of 37 °C.

**Figure 7.**
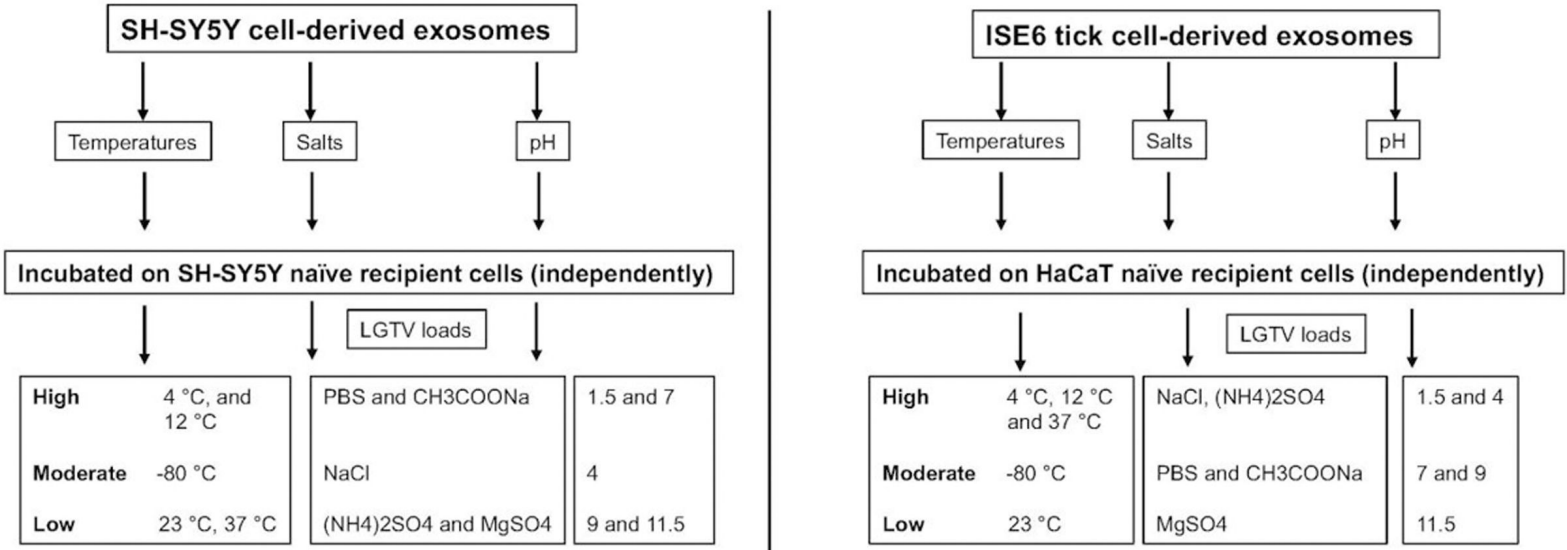
Schematic representation showing the differences with human SH-SY5Y or ISE6 tick cell-derived exosomes in relation to LGTV loads in naïve recipient SH-SY5Y host cells incubated with neuronal exosomes or human skin (HaCaT) cells incubated with tick cell-derived exosomes. Differences are shown as high, moderate or low.

Treatment of both neuronal or arthropod exosomes with MgSO_4_ showed lower viral transmission to naïve SH-SY5Y or human skin cells, respectively. In contrast to the tick-human skin model, SH-SY5Y cells also showed reduced loads when incubated with neuronal exosomes treated with (NH_4_)_2_SO_4_. This data was further confirmed with reduced NS1 protein loads in the presence of magnesium or ammonium sulfate groups. Also, we did not observe the glycosylated band for NS1 in any of the neuronal samples upon salt treatments, indicating perhaps an influence of salts on the modification of viral protein stability. Taken together, this data suggests that both magnesium/ammonium sulfates affect the transmission of flaviviruses from infectious exosomes. It has been shown that low pH increases the yield of exosome production or isolation from host cells^[81]^. Our data on neuronal or tick infectious exosomes treated at low/acidic pH (of 1.5 or 4) show increased transmission in recipient SH-SY5Y or skin cells, correlating with the above findings. Increased viral transmission and replication in naïve recipient cells may be due to increased stability of exosomes at acidic pH (of 1.5 or 4). Additionally, the consistency in reduced viral loads observed in recipient cells incubated with infectious neuronal/tick exosomes (treated at pH of 9 or 11.5) suggested that alkaline pH may inhibit/block exosome-mediated viral transmission to human cells. Our data further boost the reasons for the success of fluid-replacement therapy (with high alkaline fluids in combination with magnesium salts) that are given to dengue/Zika-infected patients with high fevers and thrombocytopenia. In addition, it is interesting to note that treatment of tick exosomes at alkaline pH (both 9 or 11.5) reduces viral transmission and replication in naïve recipient human skin cells. This observation further suggests that humans with alkaline body maintenance/topical application of solutions with high alkaline pH might get fewer tick bites and perhaps protect against the transmission of highly infectious flaviviruses. A summary of the findings from this study is shown [Figure 7]. Overall, our study represents a way to interfere with the transmission of flaviviruses and perhaps other vector-borne pathogens. We believe that this is an important study that could change the way we think about the approaches and strategies to interfere with the modes of pathogen transmission from vector to human and other vertebrate hosts.

## Supplementary Material

Supplementary

## Data Availability

All the data presented in this study are included in the article and as figures/table, further inquiries can be directed to the corresponding author.
