# Supplementary material for: Alterations in arthropod and neuronal exosomes reduce virus transmission and replication in recipient cells
Source: Extracell Vesicles Circ Nucl Acids. 2022 Aug 31;3(3):264–79. doi: 10.20517/evcna.2022.30 (PMC10018778; doi:10.20517/evcna.2022.30)
Supplement: Supplementary file 1 [file evcna-3-3-264-SupplementaryMaterials.pdf]

SUPPLEMENTARY FIGURE LEGENDS

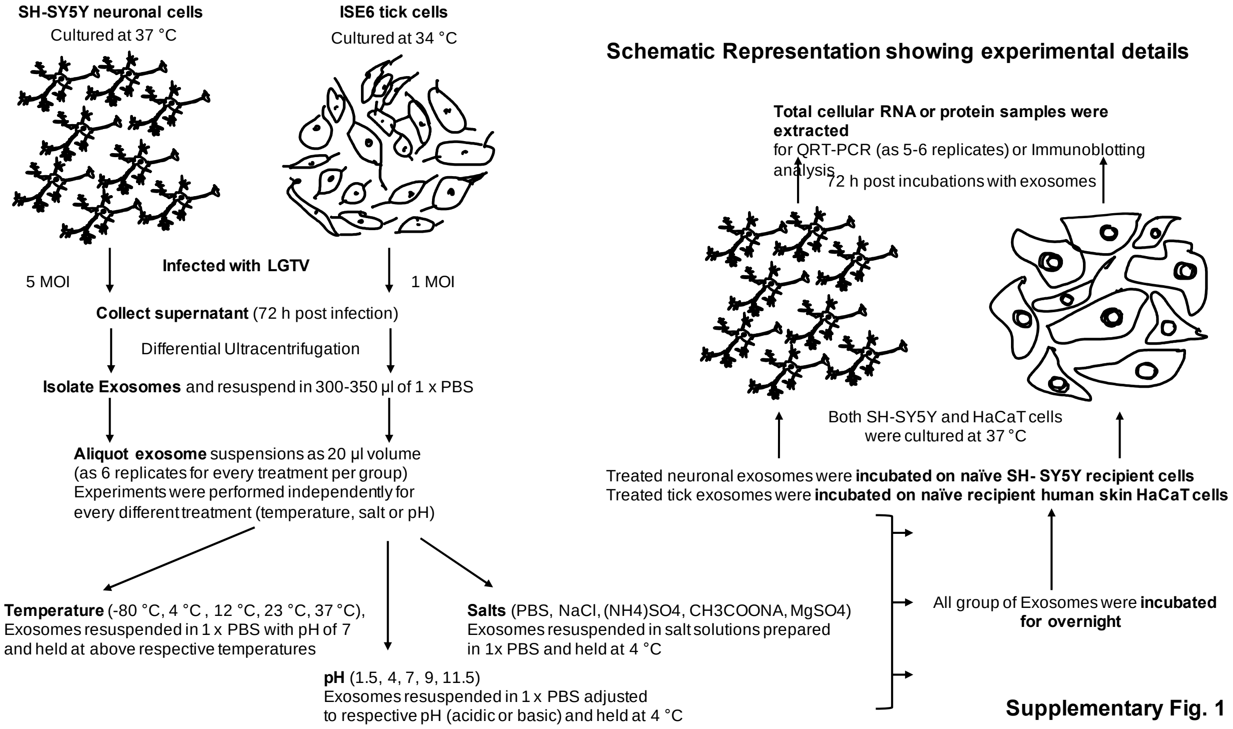

Supplementary Figure 1. Schematic showing experimental details. A schematic representation is shown for the understanding of experimental procedures used in this study. SH-SY5Y neuronal cells or ISE6 tick cells (not shown on the scale) were infected with LGTV (either 5 MOI for SH-SY5Y cells or 1 MOI for ISE6 tick cells). MOI indicates multiplication of infection. Cell culture supernatants collected after 72 h post-infection were processed for exosomes isolation by differential ultracentrifugation method. Exosomes treated with respective temperatures, salts or pH were incubated overnight at respective conditions. Naïve recipient SH-SY5Yor human skin (HaCaT) cells were incubated with respectively treated exosomes for 72 h to allow viral transmission via infectious exosomes. Total RNA/proteins were extracted and processed for qRT-PCR or immunoblotting analysis.

SH-SY5Y neuronal exosomes showing LGTV-NS1, CD9 and HSP70 levels

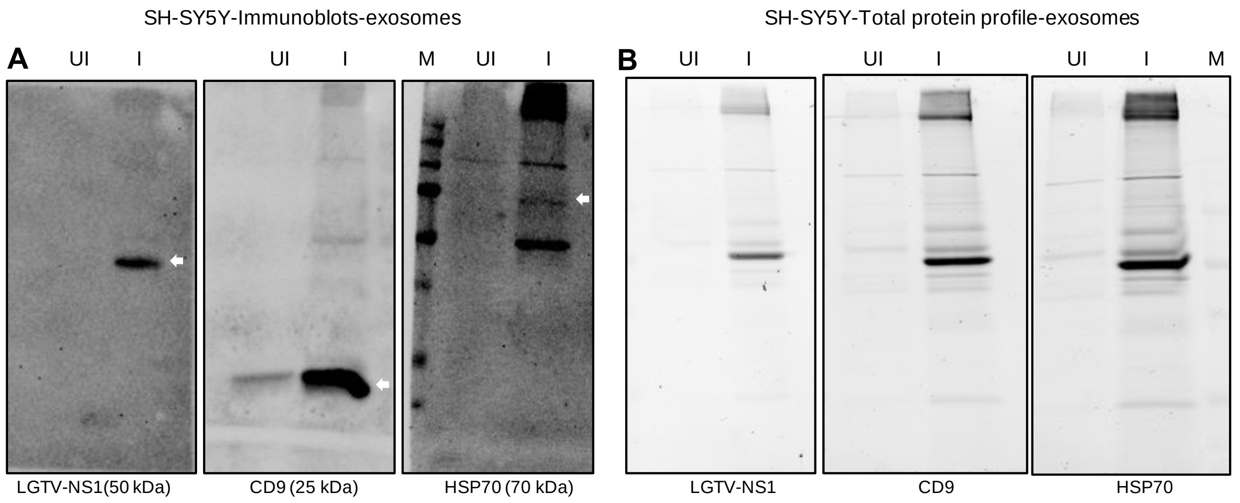

Supplementary Fig. 2

Supplementary Figure 2. Immunoblotting analysis showing exosomal markers in SH-SY5Y cells. Total protein lysates from exosomes isolated from SH-SY5Y uninfected or LGTV-infected (5 MOI) cells were probed for LGTV-NS1 protein, or CD9 or HSP70 antibodies. **A)** Immunoblots for LGTV-NS1 (50 kDa), CD9 (25 kDa) and HSP70 (70 kDa) or **B)** Total protein profiles gel images as controls are shown. Protein sizes are indicated in kilodaltons (kDa). White arrowheads indicate protein sizes.

Supplementary Table 1: Conditions used for exosome treatments is shown. Exosome suspensions treated with either different temperatures or salts or pH were held in Phosphate Buffered Saline (PBS) solution. In temperature groups, exosomes were held in 1 x PBS with neutral pH of 7 but with varying temperatures (of -80 ℃, 4 ℃, 12 ℃, 23 ℃ or 37 ℃). In salt treatment groups, exosome suspensions were prepared in 1 x PBS as 0.1 M solutions (of NaCl, (NH4)2SO4, MgSO4, CH3COONa) at pH of 7 and held at 4 ℃. In cases of pH treatment, all exosome suspensions were held in 1 x PBS solution but with varying pH (of 1.5, 4, 7, 9 or 11.5) at 4 ℃. All treatments were incubated overnight under respective conditions.

Condition of Interest	Solution	Solution pH	Overnight Storage Temperature
Temperature	PBS	7	-80 ℃
	PBS	7	4 ℃

	PBS	7	12 ℃
	PBS	7	23 ℃
	PBS	7	37 ℃
Salt	PBS	7	4 ℃
	0.1M NaCl in PBS	7	4 ℃
	0.1M $(NH_4)_2SO_4$ in PBS	7	4 ℃
	0.1M $MgSO_4$ in PBS	7	4 ℃
	0.1M CH3COONa in PBS	7	4 ℃
pH	PBS	1.5	4 ℃
	PBS	4	4 ℃
	PBS	7	4 ℃
	PBS	9	4 ℃
	PBS	11.5	4 ℃
